# 3-Bromo-4,5-Dihydroxybenzaldehyde Protects Against Myocardial Ischemia and Reperfusion Injury Through the Akt-PGC1α-Sirt3 Pathway

**DOI:** 10.3389/fphar.2018.00722

**Published:** 2018-07-10

**Authors:** Shu-Guang Qin, Hong-Yan Tian, Jin Wei, Zhen-Hua Han, Ming-Juan Zhang, Guang-Hua Hao, Xin Liu, Long-Fei Pan

**Affiliations:** ^1^Department of Cardiology, The Second Affiliated Hospital of Xi’an Jiaotong University, Xi’an Jiaotong University, Xi’an, China; ^2^Department of Cardiovascular Medicine, The First Affiliated Hospital of Xi’an Jiaotong University, Xi’an Jiaotong University, Xi’an, China; ^3^Department of Emergency Medicine, The Second Affiliated Hospital of Xi’an Jiaotong University, Xi’an Jiaotong University, Xi’an, China

**Keywords:** 3-bromo-4, 5-dihydroxybenzaldehyde, cardiomyocytes, ischemia, oxidative stress, Sirt3

## Abstract

Natural marine products are useful candidates for the treatment of oxidative and inflammatory diseases, including myocardial ischemia. 3-bromo-4,5 - dihydroxybenzaldehyde (BDB), a natural bromophenol isolated from marine red algae, has been shown to display anti-microbial, anti-oxidative, anti-cancer, anti-inflammatory, and free radical scavenging activities. In this study, the potential protective effects of BDB against myocardial ischemia and reperfusion (IR) injury was investigated in an *in vitro* model mimicked by oxygen and glucose deprivation (OGD) in cardiomyocytes and in an *in vivo* model induced by coronary artery ligation in rats. The results showed that BDB attenuated the OGD-induced cytotoxicity in a dose-dependent manner, with no toxic effect when treated alone. BDB significantly decreased apoptosis and the cleavage of caspase-3 after OGD. We found that OGD-induced oxidative stress, as evidenced by increases of reactive oxygen species (ROS) and lipid peroxidation, as well as mitochondrial dysfunction, as measured by mitochondrial reporter gene, cytochrome c release and ATP synthesis, were markedly attenuated by BDB treatment. In addition, BDB increased the enzymatic activities of mitochondrial antioxidant enzymes, including IDH2, GSH-Px and SOD2. Western blot analysis showed that BDB increased Akt phosphorylation and upregulated the expression of Sirt3 and PGC1α after OGD. Furthermore, BDB-induced protection in cardiomyocytes was partially reversed by the Akt inhibitor and downregulation of PGC1α. BDB also attenuated myocardial contractile dysfunction and activated the Akt-PGC1α-Sirt3 pathway *in vivo*. All these data suggest that BDB protects against myocardial IR injury through activating the Akt-PGC1α-Sirt3 pathway.

## Introduction

With the rising incidence of obesity and diabetes in recent years, cardiovascular disease is becoming the major cause of death worldwide. Myocardial infarction induced by coronary artery occlusion is the leading cause of morbidity and mortality for both men and women (Benjamin et al., 2018). There are approximately 100 million non-cardiac operations each year, and 3.9% of all patients experience perioperative myocardial infarction, leading to prolonged hospital stays and increased costs (Devereaux et al., 2005). Percutaneous coronary intervention (PCI) and intensive pharmacotherapy could restore blood flow and oxygen supply, but also induce damage to the previously ischemic tissue, known as reperfusion injury (Yellon and Hausenloy, 2007). To date, there is no effective therapy to prevent this form of injury.

Natural marine products are useful candidates for the identification of bioactive compounds for the treatment of oxidative and inflammatory disorders, including myocardial ischemia and reperfusion (IR) injury. Marine algae have long been used as the sources of foods, food additives and minerals, and several secondary active metabolites of marine algae are found to exert potent anti-oxidative activity (Li et al., 2011). 3-bromo-4,5-dihydroxybenzaldehyde (BDB) is a natural bromophenol isolated from marine red algae, such as *Polysiphonia morrowii*, *Rhodomela confervoides*, and *Polysiphonia urceolata* (Fan et al., 2003; Li et al., 2008; Kim et al., 2011). Previous studies have shown that BDB displays a diverse array of pharmacological activities, such as anti-microbial, anti-oxidative, anti-cancer, anti-inflammatory, and free radical scavenging activities. Researchers from Korea showed that BDB protects human HaCaT keratinocytes against ultraviolet B (UVB) radiation (Hyun et al., 2012; Piao et al., 2017). More recently, BDB was found to activate NF-E2-related factor 2 (Nrf2) and promote its localization into the nucleus, thereby enhance the level of reduced glutathione to induce anti-oxidative effects (Kim et al., 2017). In the present study, we investigated the effects of BDB on myocardial IR injury mimicked by oxygen glucose deprivation (OGD) *in vitro* or by coronary artery ligation *in vivo*, and also elucidated the potential underlying molecular mechanisms with focus on the Akt-PGC1α-Sirt3 pathway.

## Materials and Methods

### Ethics Statement

This study was carried out in accordance with the “Guide for the Care and Use of Laboratory Animals” (NIH Publication No. 85–23, revised 1996). The protocol was approved by the Xi’an Jiaotong University Committee on Ethics in the Care and Use of Laboratory Animals.

### Cell Culture and Agents

Primary culture of cardiomyocytes was prepared from 2- to 3-day-old neonatal Sprague-Dawley (SD) rats. Animals were killed by decapitation, and the hearts were excised. After removing coronary vessels using micro tweezers under optical microscope, the atria and ventricle were minced into pieces gently using micro scissors. Then the tissues were enzymatically dissociated into single cell suspension by 0.1 mg/ml collagenase I. The cells were filtered with a 70 μm cell strainer and centrifuged at 1000 rpm for 3 min to minimize fibroblast contamination. Cardiomyocytes were cultured in Dulbecco’s Modified Eagle Medium (DMEM) supplemented with 10% fetal bovine serum and incubated at 37°C in a humidified 5% CO_2_ incubator. The purity of cardiomyocytes was checked by staining with a cardiac sarcomeric α-actinin monoclonal antibody and was found to be higher than 90%. For OGD conditions, the cells were cultured in no-serum, no-glucose DMEM (Gibco) at 37°C with 1% oxygen and 5% CO_2_ in a hypoxic incubator. BDB was obtained from Matrix Scientific (Columbia, SC, United States) and dissolved in dimethyl sulfoxide (DMSO). The commercial assay kits for WST-1 assay and lactate dehydrogenase (LDH) assay and kits for measurement of enzyme activities were purchased from Jiancheng Bioengineering Institute (Nanjing, Jiangsu, China).

### WST-1 Assay

Cell viability of cardiomyocytes was determined using the WST-1 assay according to the manufacturer’s protocol. Briefly, cardiomyocytes were seeded in 96-well plates at a density of 1 × 10^4^ cells per well and exposed to OGD and/or BDB treatment. Then, 20 μL of WST-1 solution was added into each well, and the cells were incubated for further 3 h. The absorbance was measured at 450 nm, and the value of corresponding untreated control cells was set to 100%.

### LDH Release Assay

Cytotoxicity in cardiomyocytes was determined by quantifying LDH release into the culture medium according to the manufacturer’s protocol. The results were expressed as the fold of the value of corresponding untreated control cells.

### Flow Cytometry

Apoptosis in cardiomyocytes was determined using double staining with the fluorescein isothiocyanate (FITC)-conjugated Annexin V and propidium iodide (PI). Cardiomyocytes were cultured in 6-well plates and exposed to OGD and/or BDB treatment. Then, cells were harvested by trypsin digestion and washed twice with ice-cold phosphate buffered saline (PBS). Cells were stained with 5 μM Annexin V and 5 μM PI and lucifugally incubated at 37°C for 15 min. The samples were analyzed by a FACSCalibur cytometer (BD Biosciences, CA, United States), and the apoptotic rate was calculated.

### Determination of ROS Generation

Intracellular ROS generation in cardiomyocytes was detected by measuring superoxide (O^2−^) levels using DHE probe and measuring hydrogen peroxide (H_2_O_2_) levels using DCFH-DA staining as previously described (Kumar et al., 2009; Chen et al., 2012). The fluorescence intensity was detected using a Nikon fluorescence microscope, and the results were expressed as the fold of the value of corresponding untreated control cells.

### Measurement of Mitochondrial Oxidative Stress

Mitochondrial oxidative stress in cardiomyocytes was detected using a reporter gene Mito-Timer as previously described (Laker et al., 2014). After OGD and/or BDB treatment, cardiomyocytes were incubated with a mixture of Mito-Timer plasmid DNA and Lipofectamine in serum free medium (Opti-MEM medium) for 5 h. The cells were washed twice by PBS and examined by confocal microscopy at 488/518 nm (green) and 543/572 (red).

### Measurement of ATP Synthesis

Cardiomyocytes were subjected to fission and centrifuged at 12 000 *g* for 5 min. In 24-well plates, 100 μL of each supernatant was mixed with 100 μL ATP working dilution. Luminance was measured using a monochromator microplate reader. The ATP release levels were expressed as a percentage of the luminescence levels in the treated control cells.

### Measurement of Enzyme Activities

The enzymatic activities of IDH2, GSH-Px and SOD2 were measured using commercial assay kits according to the manufacturer’s instructions.

### Short Interfering RNA (siRNA) and Transfection

To knockdown the expression of Sirt3 and PGC1α protein, Si-Sirt3 (sc-61556) and Si-PGC1α (sc-72151) were obtained from Santa Cruz. Negative control siRNA Si-control (sc-37007) was used as control. The siRNA molecules were transfected using Lipofectamine RNAiMax reagent (Invitrogen, CA, United States) in Opti-MEM medium according to the manufacturer’s instructions. After incubation for 48 h, cells were treated with OGD and/or BDB.

### Myocardial IR Injury Model

Myocardial IR was induced by coronary artery ligation in rats as previously described with minor modifications (Lee et al., 2017). Rats were opened through left inter costal thoracotomy and the left anterior descending coronary artery was surgically occluded with a 6-0 suture. After 40 min of ischemia, the ligature was released to induce reperfusion. The animals were placed on a heating pad to stabilize the body temperature during anesthesia.

### *In Vivo* Experimental Design

A total of 48 male SD rats were divided into the following four groups: Sham group, BDB group, IR group and IR + BDB group. The animals in each group were subdivided into two subgroups (*n* = 6): the first subgroup was used for western blot analysis and the second subgroup was used for echocardiographic assessment. BDB (100 mg/kg) was injected via tail vein during surgery, and this dose was selected based on the literature (Kang et al., 2017).

### Echocardiographic Assessment

Echocardiographic parameters, including left ventricular end diastolic diameter (LVEDD), left ventricular end systolic diameter (LVESD), and fractional shortening (FS) were evaluated using the two-dimensional guided M-mode echocardiography (Phillips Sonos 5500) as previously described (Zhang et al., 2014).

### Western Blot Analysis

Total proteins from cardiomyocytes were extracted and the protein concentration was determined using a BCA assay kit (Jiancheng Bioengineering Institute, Jiangsu, China). Equivalent proteins (60 μg/sample) were separated using 10–12% sodium dodecyl sulfate (SDS)-PAGE, and then electro-transferred onto polyvinylidene fluoride (PVDF) membranes. The membranes were incubated with following primary antibodies: cleaved-caspase-3 (1:200), MDA (1:1000), 4-HNE (1:1000), cytochrome c (1:800), tubulin (1:2000), COX V (1:800), Sirt3 (1:500), ac-SOD2 (1:200), SOD2 (1:1000), p-Akt (1:200), Akt (1:1000), PGC1α (1:800), and β-actin (1:2000). After incubation with secondary antibodies for 1 h, the bands were visualized by using chemiluminescent detection system.

### Statistical Analysis

Each experiment was repeated at least three times. Statistical analysis was performed using SPSS. Statistical evaluation of the data was performed by one-way analysis of variance. A value of *p* < 0.05 referred to the statistical difference.

## Results

### BDB Attenuates Ischemic Injury in Cardiomyocytes

**FIGURE 1:**
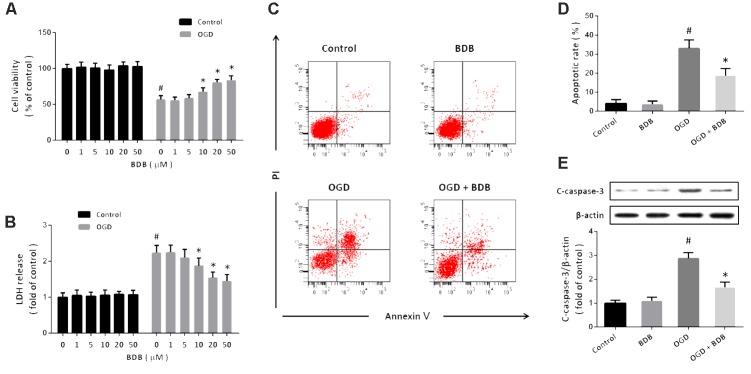
BDB attenuates ischemic injury in cardiomyocytes. **(A)** WST-1 assay shows that BDB attenuated the decrease in cell viability induced by OGD. **(B)** LDH release assay shows that BDB reduced the increase in LDH release induced by OGD. **(C,D)** Flow cytometry **(C)** and quantification **(D)** show that BDB inhibited apoptosis induced by OGD. **(E)** Western blot shows that BDB inhibited caspase-3 activation induced by OGD. Data are shown as mean ± SEM (*n* = 6). ^#^*p* < 0.05 vs. Control. ^∗^*p* < 0.05 vs. OGD.

Cardiomyocytes was treated with BDB at different concentrations to determine its potential toxicity, and BDB had no effect on cell viability (**Figure 1A**) and LDH release (**Figure 1B**) up to 50 μM. OGD induced a decrease in cell viability and an increase in LDH release, which were both significantly attenuated by BDB at 10, 20 or 50 μM, but not by BDB at 1 or 5 μM (**Figures 1A,B**). Flow cytometry was performed to detect apoptosis in cardiomyocytes (**Figure 1C**), and the results showed that 20 μM BDB reduced the apoptotic rate after OGD (**Figure 1D**). The cleavage of caspase-3 was detected by western blot using cleaved-caspase-3 antibody, and suppression of cleaved-caspase-3 expression was observed after BDB treatment (**Figure 1E**).

### BDB Reduces Oxidative Stress After Ischemia

**FIGURE 2:**
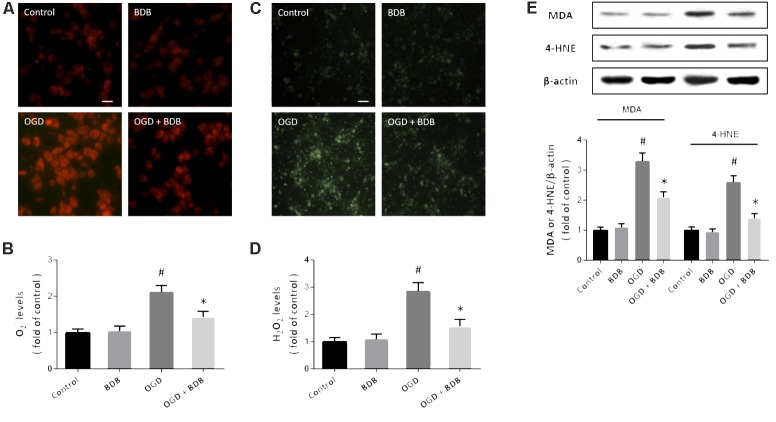
BDB reduces oxidative stress after ischemia. **(A,B)** DHE staining **(A)** and quantification **(B)** show that BDB reduced the increase in O^2−^ levels induced by OGD. **(C,D)** DCFH-DA staining **(C)** and quantification **(D)** show that BDB reduced H_2_O_2_ levels induced by OGD. **(E)** Western blot shows that BDB attenuated lipid peroxidation induced by OGD. Scale bar, 20 μm. Data are shown as mean ± SEM (*n* = 6). ^#^*p* < 0.05 vs. Control. ^∗^*p* < 0.05 vs. OGD.

Oxidative stress has been demonstrated to be involved in the pathogenesis of myocardial IR. Intracellular ROS generation was detected by measuring O^2−^ levels using DHE probe (**Figure 2A**) and measuring H_2_O_2_ levels using DCFH-DA staining (**Figure 2C**). OGD resulted in significant increases in both O^2−^ and H_2_O_2_ levels, which were attenuated by 20 μM BDB treatment (**Figures 2B,D**). We also performed western blot using the MDA and 4-HNE antibodies to detect lipid peroxidation, and the results showed that increased expression of MDA and 4-HNE after OGD were markedly decreased by BDB (**Figure 2E**).

### BDB Preserves Mitochondrial Function After Ischemia

**FIGURE 3:**
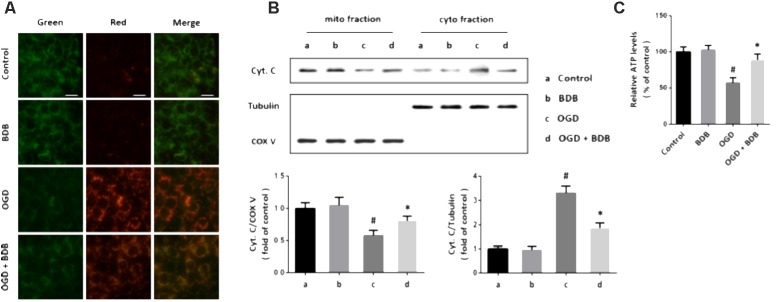
BDB preserves mitochondrial function after ischemia. **(A)** Mito-Timer reporter gene assay shows that BDB reduced mitochondrial oxidative stress induced by OGD. **(B)** Western blot shows that BDB attenuated cytochrome c release from mitochondria induced by OGD. **(C)** ATP synthesis assay shows that BDB preserved mitochondrial ATP generation after OGD. Scale bar, 20 μm. Data are shown as mean ± SEM (*n* = 6). ^#^*p* < 0.05 vs. Control. ^∗^*p* < 0.05 vs. OGD.

A novel reporter gene was used to detect mitochondrial oxidative stress in cardiomyocytes, and an increase of red fluorescence intensity and decrease of green fluorescence intensity was observed after OGD (**Figure 3A**). Following BDB treatment, the red/green ratio was markedly reduced compared to that in the OGD group. Next, we performed western blot using cytochrome c antibody in mitochondrial and cytosolic fractions to determine cytochrome c release. As shown in **Figure 3B**, an increase in cytosolic cytochrome c level and a decrease in mitochondrial cytochrome c level were found in OGD-injured cells, indicating cytochrome c release after OGD. Treatment with BDB significantly inhibited the cytochrome c release induced by OGD. In addition, BDB treatment also preserved mitochondrial ATP generation after OGD in cardiomyocytes (**Figure 3C**).

### BDB Activates Sirt3-Mediated Mitochondrial Enzyme Activities

**FIGURE 4:**
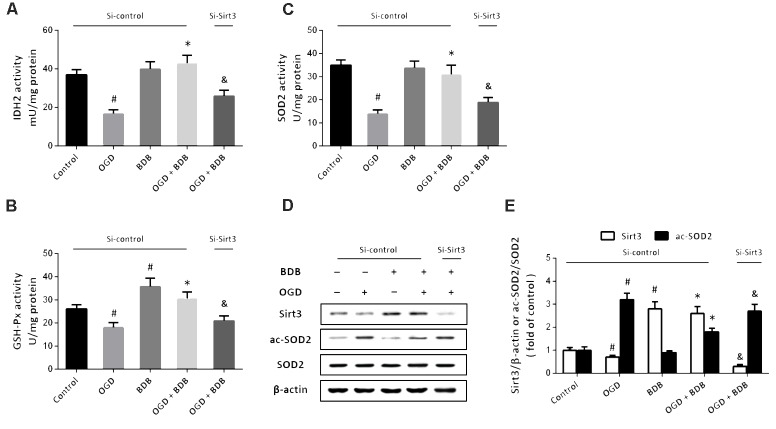
BDB activates Sirt3-mediated mitochondrial enzyme activities. **(A)** Quantification shows that BDB increased IDH2 activity after OGD. **(B)** Quantification shows that BDB increased GSH-Px activity after OGD. **(C)** Quantification shows that BDB increased SOD2 activity after OGD. **(D,E)** Western blot **(D)** and quantification **(E)** show that BDB increased Sirt3 expression and SOD2 deacetylation after OGD. Data are shown as mean ± SEM (*n* = 6). ^#^*p* < 0.05 vs. Control. ^∗^*p* < 0.05 vs. OGD. ^&^*p* < 0.05 vs. Si-control.

To investigate the mechanisms underlying BDB-induced anti-oxidative effects, the enzymatic activities of IDH2, GSH-Px and SOD2 were measured. As shown in **Figures 4A–C**, the activities of these enzymes were significantly reduced by OGD insult. Treatment with 20 μM BDB promoted these enzymes activities compared to OGD group. We performed western blot to detect the acetylation of SOD2 (**Figure 4D**), and the results showed that OGD increased the acetylation of SOD2, which was partially reversed by BDB (**Figure 4E**). In addition, BDB treatment significantly increased the expression of Sirt3 both in the presence and absence of OGD (**Figures 4D,E**). We repeated these experiments after downregulating Sirt3 expression using siRNA transfection, and the results showed that BDB-induced effects on enzyme activities and SOD2 acetylation were prevented by Si-Sirt3.

### BDB Stimulates the Akt-PGC1α-Sirt3 Pathway in Cardiomyocytes

**FIGURE 5:**
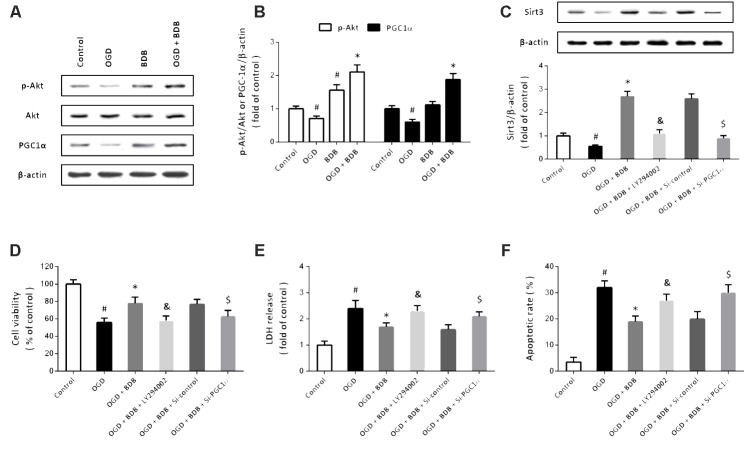
BDB stimulates the Akt-PGC1α-Sirt3 pathway in cardiomyocytes. **(A,B)** Western blot **(A)** and quantification **(B)** show that BDB increased PGC1α expression and Akt phosphorylation after OGD. **(C)** Western blot shows that BDB-induced Sirt3 expression was dependent on Akt and PGC1α. **(D)** WST-1 assay shows that BDB-induced increase in cell viability after OGD was dependent on Akt and PGC1α. **(E)** LDH release assay shows that BDB-induced decrease in LDH release after OGD was dependent on Akt and PGC1α. **(F)** Flow cytometry shows that BDB-induced inhibition in apoptosis after OGD was dependent on Akt and PGC1α. Data are shown as mean ± SEM (*n* = 6). ^#^*p* < 0.05 vs. Control. ^∗^*p* < 0.05 vs. OGD. ^&^*p* < 0.05 vs. OGD + BDB. ^$^*p* < 0.05 vs. Si-control.

Then, western blot was performed to detect the expression of Akt and PGC1α, two important upstream factors of Sirt3 signaling (**Figure 5A**). The results showed that OGD inhibited the phosphorylation of Akt and PGC1α expression, which were both partially prevented by BDB (**Figure 5B**). To further investigate the involvement of Akt and PGC1α, cardiomyocytes were transfected with PGC1α targeted siRNA to knockdown PGC1α or treated with LY294002 to block Akt activation. Western blot showed that BDB-induced increase in Sirt3 expression after OGD was attenuated by LY294002 and Si-PGC1α transfection (**Figure 5C**). Furthermore, BDB-induced protection against OGD, as measured by cell viability (**Figure 5D**), LDH release (**Figure 5E**) and apoptosis (**Figure 5F**), were all reduced by Akt or PGC1α inhibition.

### Cardioprotective Effects of BDB *in Vivo*

**FIGURE 6:**
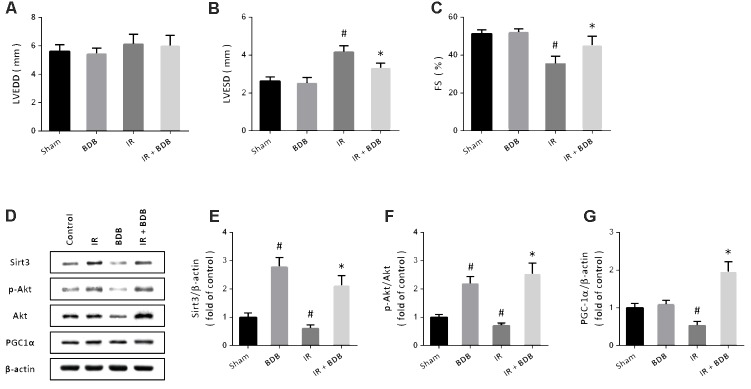
Cardioprotective effects of BDB *in vivo*. **(A–C)** Echocardiographic parameters show that BDB had no effect on LVEDD **(A)**, but decreased LVESD **(B)** and increased FS **(C)** after myocardial IR in rats. **(D–G)** Western blot **(D)** and quantification show that BDB increased the expression of Sirt3 **(E)** and PGC1α **(F)** and Akt phosphorylation **(G)** after myocardial IR in rats. Data are shown as mean ± SEM (*n* = 6). ^#^*p* < 0.05 vs. Sham. ^∗^*p* < 0.05 vs. IR.

To investigate whether BDB has cardioprotective effects *in vivo*, a myocardial IR model induced by coronary artery ligation was performed in rats. Echocardiographic parameters showed that BDB significantly decreased LVESD (**Figure 6B**) but increased FS (**Figure 6C**) after myocardial IR in rats. BDB treatment had no effect on LVEDD in rats with or without myocardial IR (**Figure 6A**). In addition, western blot was used to detect the effects of BDB on Akt-PGC1α-Sirt3 pathway *in vivo* (**Figure 6D**). As expected, the results showed that BDB markedly increased the expression of Sirt3 (**Figure 6E**) and PGC1α (**Figure 6F**) and Akt phosphorylation (**Figure 6G**) after myocardial IR in rats.

## Discussion

Myocardial infarction is the leading cause of death worldwide, and it is highly desirable to develop safe and effective drugs to reduce cardiomyocytes death during IR. Our present results provide evidence that BDB, a natural bromophenol compound isolated from red algae, protects against myocardial IR *in vitro* and *in vivo*. We found that (a) BDB promotes cell survival and inhibits apoptosis after OGD; (b) BDB attenuates OGD-induced oxidative stress and mitochondrial dysfunction; (c) BDB activates antioxidant enzymes and decreases acetylation of SOD2; (d) BDB promotes phosphorylation of Akt and expression of Sirt3 and PGC1α; (e) blocking the Akt-PGC1α-Sirt3 pathway partially abolished the protective effects of BDB in cardiomyocytes, and (f) BDB attenuated myocardial contractile dysfunction and activated Akt-PGC1α-Sirt3 pathway *in vivo*.

Bromophenols extracted from marine plants have been demonstrated to exert many biological activities, including anti-oxidative, anti-bacterial, anti-inflammatory and anti-cancer activities (Li et al., 2011; Liu M. et al., 2011). BDB is a bromophenol compound isolated from marine red algae with novel pharmaceutical applications and nutritional values. It was found to exhibit anti-viral activities with the 50% effect concentration (EC_50_) of 45 μM against both infectious hematopoietic necrosis virus and infectious pancreatic necrosis virus (Kim et al., 2011). A previous study showed that 10 μM BDB significantly increased cell viability of human keratinocytes after UVB radiation (Piao et al., 2017). Kang et al. demonstrated that in murine macrophages, BDB at the concentration ranging from 12.5 to 100 μM inhibited the production of inflammatory cytokines in a dose-dependent manner (Kang et al., 2017). Our results showed that BDB at 10, 20, and 50 μM significantly decreased OGD-induced cytotoxicity in cardiomyocytes, extending the protective effects of BDB into cardiovascular disease conditions. Piao et al. (2017) previously found that BDB was not toxic to human keratinocytes up to 30 μM, but showed cytotoxicity at 40 and 50 μM. Intriguingly, no obvious cytotoxic effect was found when cardiomyocytes were treated with 50 μM BDB in our *in vitro* conditions. In addition, we also observed protective effects of BDB against myocardial contractile dysfunction in rats. These results indicate that BDB might be an ideal cytoprotective agent with low toxicity, which depends on cell types.

Mitochondria are the sites of oxidative metabolism, where the life-supporting energy is generated via the production of ATP. Due to high energetic requirements of the heart, cardiomyocytes have high mitochondrial densities and nearly one-third of the cell’s volume is occupied by mitochondria (Moreno-Lastres et al., 2012). Thus, cardiomyocytes are particularly susceptible to mitochondrial toxins, and mitochondrial dysfunction has been demonstrated as a key event in myocardial injury during IR (Lesnefsky et al., 2017). Damage to cardiac mitochondria impaired energy generation through decreasing the rates of substrate oxidation, destroying the phosphorylation apparatus, impairing the creatine shuttle and blocking the export of high energy phosphates from mitochondria (Lesnefsky et al., 2001). In cardiomyocytes, mitochondria are the main sources of ROS, which can cause cardiac hypertrophy, fibrosis, contractile dysfunction and heart failure (Seddon et al., 2007). In this study, OGD exposure caused significant increases in O^2−^ and H_2_O_2_ levels, which were attenuated by BDB treatment. Thus, we further measured mitochondrial oxidative stress and cytochrome c release to investigate its effect on mitochondrial function. The results demonstrated that BDB markedly attenuated mitochondrial dysfunction after OGD. Endogenous antioxidants, such as SOD2, catalase and GSH-Px, are the first line of defense against oxidative stress (Rashid et al., 2013). Ischemia inhibits activities of these mitochondrial enzymes, which further promotes oxidative stress and accelerates cardiomyocytes death (Dhalla et al., 1999). In this study, BDB was found to stimulate the enzymatic activities of IDH2, GSH-Px and SOD2 both in the presence and absence of OGD. All these data indicated that BDB-induced protection in cardiomyocytes were associated with preservation of mitochondrial function and suppression of oxidative stress.

The sirtuins are a family of nicotinamide adenine dinucleotide (NAD^+^) -dependent class III histone deacetylases, which also have many non-histone protein targets and functions (Liu Y. et al., 2011). Mammals express seven homologs of sirtuins, Sirt1-7, among which Sirt3 is unique due to its location in mitochondria and involvement in extending human lifespan (Kincaid and Bossy-Wetzel, 2013). Sirt3 is highly expressed in organs with high energetic requirements and mitochondrial content, such as brain, liver and heart (Dai et al., 2017). In rodents, increased expression of Sirt3 in cardiomyocytes was found in the early phase of cardiovascular disorders, but Sirt3 expression was decreased in human failing hearts (Grillon et al., 2012; Parodi-Rullan et al., 2012). In our *in vitro* model, decreased expression of Sirt3 was observed in cardiomyocytes following OGD, which was accompanied by increased apoptosis. Thus, the downregulation of Sirt3 might be maladaptive and may exaggerate cell damage in cardiomyocytes under oxidative stress. Cardiomyocytes cultured from mice with global deletion of Sirt3 showed increased ROS levels compared to the wide-type controls, and overexpression of Sirt3 exerted myocardial protective effects (Pillai et al., 2010). A previous study showed that Sirt3 deficiency increased myocardial infarct size after ischemia and impaired the recovery of contractile function following reperfusion (Porter et al., 2014). Our result showed that BDB increased Sirt3 expression both *in vitro* and *in vivo*, and BDB-induced protection was partially abolished after Sirt3 knockdown, indicating the involvement of Sirt3. Sundaresan et al. (2008) demonstrated that overexpression of Sirt3 could block the translocation of Bax into mitochondria and thereby inhibit apoptosis. All these results suggested that Sirt3-mediated anti-oxidative and anti-apoptotic mechanisms contributed to the protective effects of BDB against myocardial IR injury.

Further experiments were performed to investigate the upstream factors that regulate Sirt3 after BDB treatment in cardiomyocytes. Western blot analysis showed increased expression of PGC1α and increased phosphorylation of Akt, even in the absence of OGD exposure. PGC1α belongs to the PGC1 family, which are key regulators of mitochondrial biogenesis and metabolism (Liu and Lin, 2011; Scarpulla, 2011). It was originally identified in brown adipose tissue, and has been shown to be highly expressed in cells with high oxidative capacity, such as neurons and cardiomyocytes (Puigserver et al., 1998). The expression of PGC1α was shown to be inhibited in various heart failure models (Rowe et al., 2010), which was also observed in our *in vitro* and *in vivo* IR injury model. PGC1α could activate many transcription factors to regulate gene expression of both nuclear and mitochondrial proteins, including Sirt3 (Kong et al., 2010; Shi et al., 2015; Chen et al., 2018). Our present study confirmed this by the result that BDB-induced expression of Sirt3 was partially prevented by PGC1α knockdown. Akt, also known as protein kinase B, is another upstream factor that can regulate both Sirt3 and PGC1α. A previous study in hepatocytes found that Akt directly phosphorylated PGC1α and inhibited the recruitment of PGC1α to its cognate promoter regions (Li et al., 2007). FGF21 was shown to protect against diabetes-induced apoptosis via upregulating Akt and PGC1α, and Akt and PGC1α activation contributed to phosphodiesterase-5 inhibitor-induced preservation of mitochondrial function in type 2 diabetic hearts (Koka et al., 2014; Jiang et al., 2015). More recently, upregulation of Sirt3 induced by melatonin was shown to attenuate hepatotoxicity through activating PI3K/Akt-PGC1α pathway (Song et al., 2017). We found that blocking Akt and PGC1α activation not only attenuated BDB-induced expression of Sirt3, but also partially reversed BDB-induced protection against OGD. All these data strongly support that BDB protects cardiomyocytes against ischemia through activating the Akt-PGC1α-Sirt3 pathway.

There was one limitation to our present study. Our results showed that BDB significantly reduced O^2−^ and H_2_O_2_ levels after OGD, which might be associated with the Sirt3-mediated activation of mitochondrial enzymes. However, many other ROS buffering signaling cascades are also involved in oxidative stress in myocardial IR injury. For example, the mitochondrial peroxidase peroxiredoxin-3 reduces H_2_O_2_ to H_2_O using reducing equivalents from NADPH supplied by thioredoxin-2 (Trx2) and thioredoxin reductase-2 (TrxR2) in isolated heart mitochondria (Stanley et al., 2011). Whether BDB exerts protective effects via regulating the TrxR2/Trx2 system needs to be determined in the future.

In summary, this study suggests that BDB, a natural bromophenol found in seaweeds, protects against myocardial IR injury via activating the Akt-PGC1α-Sirt3 pathway. These findings may reveal a new feature for the mechanism of BDB in cardioprotective effects.

## Author Contributions

S-GQ and L-FP conceived and designed the experiments. S-GQ, H-YT, JW, M-JZ, and G-HH performed the experiments. XL and Z-HH analyzed the data. S-GQ and L-FP wrote the paper.

## Conflict of Interest Statement

The authors declare that the research was conducted in the absence of any commercial or financial relationships that could be construed as a potential conflict of interest.
